# Particularités radiologiques de la tuberculose pulmonaire à microscopie positive au Service de Pneumologie du Centre Hospitalier National Universitaire de FANN (CHNUF), Dakar (Sénégal)

**DOI:** 10.11604/pamj.2018.30.21.14208

**Published:** 2018-05-09

**Authors:** Ulrich Davy Kombila, Yacine Dia Kane, Fatimata Bintou Rassoule Mbaye, Ndeye Fatou Diouf, Waly Ka, Nafissatou Oumar Touré

**Affiliations:** 1 Service de Médecine Interne, Centre Hospitalier Universitaire de Libreville (CHUL), Libreville, Gabon; 2 Clinique de Pneumologie, Centre Hospitalier National Universitaire de FANN (CHNUF), Dakar, Sénégal

**Keywords:** Anomalies radiologiques, tuberculose pulmonaire, BAAR positif, Dakar, Radiological abnormalities, pulmonary tuberculosis, AFB positive, Dakar

## Abstract

L’objectif était de déterminer les différents aspects radiologiques de la tuberculose pulmonaire à microscopie positive. Il s’agissait d’une étude rétrospective, analysant les clichés de radiographies des patients atteints de tuberculose pulmonaire à microscopie positive entre le 15 Novembre 2015 et le 15 Mars 2016. Soixante-six dossiers répondant aux critères d’inclusion ont été sélectionnés, composé de 81,8% des hommes. La moyenne d’âge était de 37,5 ± 14,9 ans. Les différentes lésions parenchymateuses, étaient variables et dominées par les infiltrats dans 84,8% (IC95%; 73,9 – 92,4%) des cas, suivi du syndrome alvéolaire dans 68,2% (IC95%; 55,5 – 79,1%) des cas. Elles étaient extensives dans 71,2% (IC95%; 58,7 – 81,7%) des cas et bilatérales dans 45,4% (IC95%; 31,1 – 58,1%) des cas. Ces anomalies radiologiques survenaient dans 52,2% (IC95%; 36,9 – 67,1%) des cas chez les patients dénutrie. Dans 22,7% (IC95%; 13,3 – 34,7%) des cas il s’agissait d’un second épisode de tuberculose pulmonaire. La consommation régulière de tabac a été retrouvée chez 34,8% (IC95; 23,5 – 47,5%) des patients avec une consommation moyenne de 17PA (±11,3). Les rechutes tuberculeuses étaient plus fréquentes chez les patients qui avaient fumés versus les non-fumeurs respectivement dans 26,1% et 20,9% (p < 0,42). La sérologie rétrovirale profil 1, était positive dans 7,6% des cas. A travers cette étude, nous préconisons en zone d’endémie tuberculeuse d’orienter la démarche étiologique vers la recherche d’une tuberculose pulmonaire devant des images radiologiques infiltratives associées ou non à des lésions cavitaires chez un patient jeune, dénutrie et à fortiori fumeur.

## Abstract (en)

This study aimed to determine the radiological features of microscopy-positive pulmonary tuberculosis (TB). We conducted a retrospective study, analyzing radiographical imaging of patients with microscopy-positive pulmonary tuberculosis between 15 November 2015 and 15 March 2016. The medical records of 66 patients meeting the inclusion criteria were selected, 81.8% of which belonged to men. The average age of patients was 37.5 ± 14.9 years. Patients had variable parenchymal lesions dominated by infiltrated lesions in 84.8% (CI 95%; 73.9-92.4%) of cases, followed by alveolar syndrome in 68.2% (CI95%; 55.5-79.1%) of cases. Lesions were extensive in 71.2% (CI 95%; 58.7-81.7%) of cases and bilateral in 45.4% (CI 95%; 31.1-58.1%) of cases. These radiological abnormalities occurred in 52.2% (CI 95%; 36.9 - 67.1%) of cases in undernourished patients. In 22.7% (CI 95%; 13.3-34.7%) of cases, these lesions were secondary to an episode of pulmonary tuberculosis; 34.8% (IC95; 23.5 - 47.5%) of patients were regular tobacco smokers consuming, on average, 17 tobacco packs per year (±11.3). TB recurrences were more frequent in patients with a history of smoking versus non-smokers (26.1% versus 20.9%, p < 0.42 respectively). HIV-1 serology test was positive in 7.6% of cases. This study highlights the importance of suspecting pulmonary tuberculosis in young undernourished patients, having a history of smoking, with infiltrated lesions associated or not with cavitary lesions on radiographic examination and living in TB epidemic area.

## Introduction

La tuberculose pulmonaire est une cause majeure de morbi-mortalité dans le monde. Elle est responsable du plus grand nombre de décès causés par un agent infectieux [1], et sévit sous un mode endémique dans les pays en développement. Au Sénégal, la tuberculose reste une préoccupation majeure des autorités sanitaires. Selon l’OMS [2], le Sénégal en 2015 avait déclaré 13.599 cas de tuberculose avec 84% des cas confirmé bactériologiquement parmi les cas pulmonaires. La radiographie thoracique reste l’imagerie de première intention. Les manifestations radiologiques de la tuberculose peuvent varier selon des facteurs liés à l’hôte, mais dans la plupart des cas elles sont suffisamment caractéristiques pour suggérer le diagnostic. Bien que la TDM soit généralement requise pour détecter les lésions de petites tailles invisibles sur les radiographies standards, préciser les aspects équivoques ou analyser les complications [3], elle n’est pas toujours d’accès facile dans les pays à ressources limitées. La confrontation radio-clinique reste la démarche diagnostic habituelle, mais sa confirmation ne peut être que bactériologique et/ou histologique sans que cette dernière ne soit spécifique [4]. C’est pourquoi il nous est apparu opportun de réaliser ce travail dont le but était de déterminer les différents aspects radiologiques de la tuberculose pulmonaire à microscopie positive afin de contribuer à l’amélioration de sa prise en charge précoce.

## Méthodes

Il s’agit d’une étude rétrospective, par analyse des dossiers de patients hospitalisés au service de pneumologie du centre hospitalier universitaire de FANN (CHNUF) durant la période d’activité du service allant du 15 Novembre 2014 au 15 Mars 2016, soit 4 mois. La taille de l’échantillon a été l’effectif des patients répondant aux critères d’inclusion, soit 66 patients. Ces critères étaient les dossiers de patients âgé de 15 ans et plus quelque soit le sexe dont le diagnostic à la sortie était une tuberculose pulmonaire à microscopie positive. Tous les dossiers de patients ne comportant pas une radiographie thoracique de face interprétable et/ou le diagnostic de sortie était une tuberculose extra-pulmonaire n’ont pas été retenus. Pour l’analyse des lésions anatomoradiologiques nous avons procédé de la manière suivante: la distance séparant la coupole diaphragmatique de l’apex a été divisée en trois tiers. Le tiers supérieur est situé au-dessus du 2^ème^ arc costal antérieur, le tiers moyen est située entre les 2^ème^ et 4^ème^ arcs costaux antérieurs et le tiers inférieur entre le 4^ème^ arc costal antérieur et le diaphragme. Les lésions pulmonaires ont été considérées comme extensives lorsqu’elles intéressaient au moins les 2/3 d’un hémithorax et non extensives quand elles n’excédaient pas le 1/3 de l’hémithorax concerné. Les lésions étaient considérées comme associées lorsqu’en plus des lésions parenchymateuses on notait des adénopathies médiastinales, des épanchements pleuraux (quelque soit la nature). Ces données ont été analysées grâce au logiciel Epi info 7 (version 7.2.0.1.). Les variables quantitatives étaient représentées par leur moyenne ± écart type (ET) et les données qualitatives étaient représentées avec leurs pourcentages et leur intervalle de confiance à 95%. Le test de Chi2 a été utilisé par les comparaisons des variables et corrigé selon Yates ou Fisher en fonction des effectifs des sous groupes au seuil de signification de 5%.

## Résultats

### Résultats généraux (Tableau 1)

Durant la période d’étude il avait été hospitalisé 217 patients, et 66 d’entre eux avaient développés une tuberculose pulmonaire à microscopie positive, soit une fréquence hospitalière des TPM+ de 30,4%. La population de l’étude était constituée de 81,8% (n = 54) des hommes et 18,2% (n = 12) des femmes, soit un sex ratio de 4,5. L’âge moyen des patients était de 37,5 ± 14,9 ans (extrêmes: 18 et 79 ans) avec un pic de fréquence dans la tranche de 15 à 34 ans représentant 41,1% de notre effectif. Près de la moitié des patients: 43,9% (IC95%; 31,7 – 56,7%) exerçaient dans le secteur informel et 59,1% (IC95%; 46,2 – 71%) de la population de l’étude vivait en dessous du seuil de pauvreté attesté par un revenu ou un salaire qui était en dessous du salaire minimum interprofessionnel garanti (SMIG). La notion de tabagisme actif était retrouvée chez 34,8% (IC95%; 23,5 – 47,5%) des patients. La majorité des patients; 43,9% (IC95%; 31,7 – 56,7%), et 42,4% (IC95%; 30,3 – 55,2%) provenait respectivement de Dakar et de la banlieue.

**Tableau 1:** Caractéristiques sociodémographiques des patients de l'étude (n=66)

	Fréquence (n=66)	Pourcentage (%)
***Age (ans)***		
15 – 24	11	16,6
25 – 34	27	41,1
35 – 44	7	10,6
45 – 54	13	19,7
55 – 64	4	6,0
≥ 65	4	6,0
***Sexe***		
Hommes	54	81,2
Femmes	12	18,2
***Profession***		
Etudiant/Elève	6	9,1
Retraité	5	7,6
Salarié	8	12,1
Sans profession	18	27,3
Secteur informel	29	43,9
*Situationtabagique*		
Fumeurs actuels	23	34,8
Non-fumeurs	43	65,2
***Situation*maritale**		
Célibataire	32	48,5
Marié	32	48,5
Divorcé	2	3,0
Veuve (fe)	0	0,0
Revenu < SMIG*		
Oui	39	59,1
Non	27	40,9

* SMIG= Salaire Minimum Interprofessionnel Garanti qui est de 48000 F CFA (74€ ou 77$) au Sénégal (N.O. Touré, 2011 (6)

### Antécédents

L’anamnèse avait retrouvé les antécédents de tuberculose dans 22,7% (IC95; 13,3 – 34,7%) des cas. Les rechutes tuberculeuses étaient plus fréquentes dans 26,1% des cas chez les patients fumeurs versus 20,9% des cas des patients non-fumeurs, sans différence statistiquement significative (OR = 1,3 (IC95; 0,4 – 4,3%); p < 0,42). Parmi les 66 patients, 34,8% déclaraient fumer régulièrement, au moment de leur hospitalisation, et tous étaient de sexe masculin avec une consommation moyenne de 17 PA (±15,3). La moyenne d’âge du début du tabagisme était 18,9 (±5,7) ans. Près de la moitié (47,8%) des patients consommaient entre 1 et 10 PA. La proportion des patients qui fumait entre 11 et 20 PA et plus de 20 PA était respectivement de 26,1% chacun. La consommation de substances psycho-actives chez patients fumeurs telles que le chanvre indien était retrouvée chez 15,2% (IC95%; 7,5 – 26,1%) des cas et l’alcool dans 12,2% (IC95%; 5,3 – 22,4%) des cas. La présence des comorbidités était notée dans 15,2% (IC95%; 7,5 – 26,1%) des cas et était essentiellement représentée par le VIH_1_ (7,6%). Le diabète était représenté dans 6,1% des cas dont 4,5% de type 1 et 1,6% de type 2. Le portage de l’AgHBs était retrouvé chez un seul patient. Seul un patient avait présenté l’association de plusieurs comorbidités (AgHBs positif, VIH_1_ positif).

### Données cliniques

Le délai moyen de prise en charge était de 111,8 ± 108,6 jours. Les trois quart de la population d’étude avaient consulté 180 jours après le début de la symptomatologie, soit 6 mois. Ce délai médian de prise en charge, était plus important chez les femmes que chez les hommes respectivement: 155 ± 135,8 jours contre 102,2 ± 100,5 jours sans différence statistiquement significative entre les deux genres (p<0,10). L’index de masse corporelle (IMC) n’était disponible que pour 46 patients. La moyenne de l’IMC était de 17,3 ± 3,3kg/m^2^. La majorité (52,2%) des patients était en état de dénutrition jugé par un IMC < 16,5kg/m^2^ (Tableau 2).

**Tableau 2:** Répartition de l'index de masse corporelle (IMC) par classe de la population de l'étude (n = 46)

	Fréquence (n)	Pourcentage (%)	
***Classe d’IMC (kg/m^2^)***			***Interprétation***
< 16,5	24	52,2	Dénutrition
16,5 – 18,5	6	13,0	Amaigrissement
18,5 – 25	15	32,6	Poids normal
25 – 30	1	2,2	Surpoids
> 30	0	0,0	Obésité
**Total**	46	100	

IMC = Index de Masse Corporelle

### Données biologiques

Quarante-neuf patients avaient réalisés un hémogramme, dont les résultats de la formule leucocytaire était: hyperleucocytose (28,6%), leucopénie (6,1%), lymphopénie (81,6%). Le taux moyen d’hémoglobine était de 11,2 g/dl (±3,2). Une anémie microcytaire hypochrome était retrouvée dans 63,3% des cas.

### Anomalies anatomo-radiographiques

**Figure 1:**
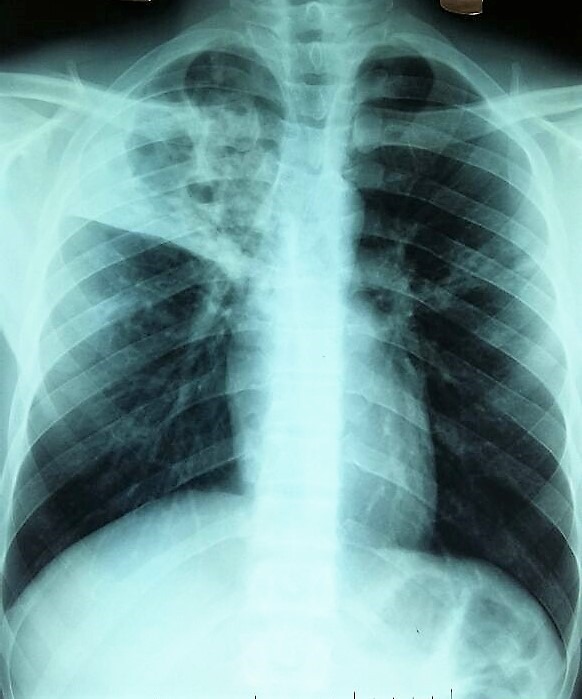
Radiographie thoracique de face: l’orbite supérieure droite poly-excavée dépassée en homolatérale et controlatérale (opacités infiltratives et micronodulaire hilo-axillaire gauche)

**Figure 2:**
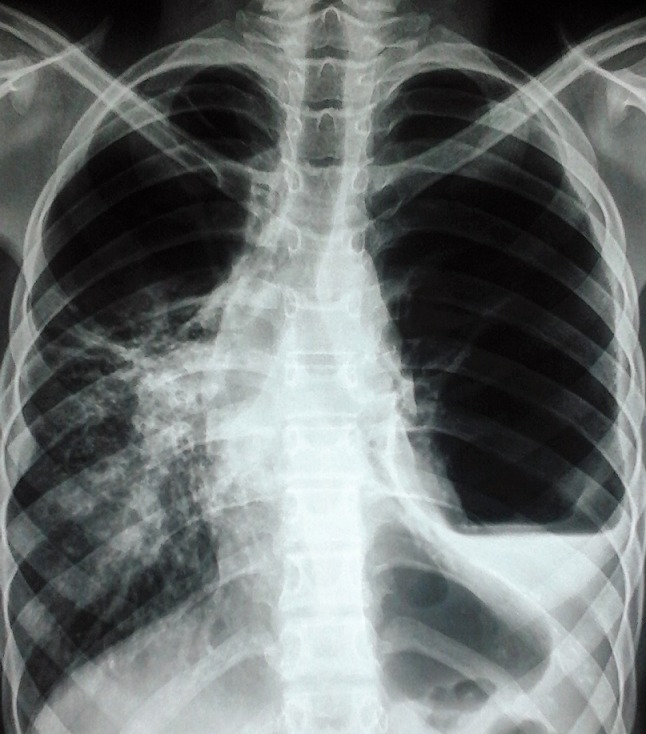
Radiographie thoracique de face aspect du poumon gauche détruit; hémithorax droit: évidement (pneumatocel) du 1/3 supérieur associé à des opacités hétérogènes d’âges différents rétractiles intéressant les 2/3 inférieurs avec déformation du hile et de la courbure de la coupole diaphragmatique

Les lésions radiologiques étaient à prédominance bilatérale dans 45,5% (IC95%; 33,1 – 58,1%) des cas et extensives dans 71,2% (IC95%; 58,7 – 81,7%). Les infiltrats étaient les lésions élémentaires radiologiques les plus représentatives dans 84,8% (IC95%; 73,9 – 92,4%) des cas (Tableau 3). Les anomalies radiologiques particulières étaient par ordre de fréquence: la lobite supérieure excavée (7,6%) (Figure 1), l’aspect de poumon détruit (4,5%) (Figure 2), siégeant dans la majorité des cas à gauche, la miliaire bronchogène (3,0%) et le pyothorax à droite, associé à une atteinte parenchymateuse (3,0%). On avait également noté une miliaire hématogène (1,5%) et une greffe aspergillaire (1,5%). Lorsque les lésions étaient unilatérales, elles intéressaient plus souvent le 1/3 supérieur.

**Tableau 3:** Caractéristiques des lésions anatomoradiologiques (n=66)

	Fréquence (N)	Pourcentage (%)
*Siège des lésions (n=66)*		
Bilatérale	30	45,5
Hémithorax droit	20	30,3
Hémithorax gauche	16	14,2
*Anomalies radiologiques*		
Cavernes	39	59,1
Infiltrats	56	84,8
Sd. alvéolaire	46	68,2
*Lésions associées*		
ADP médiastinales	4	6,1
Pleurésies	6	9,1

Sd. = syndrome; ADP = Adénopathie(s)

## Discussion

Nous avons été confrontés à certaines difficultés inhérentes aux études rétrospectives. Il s’agit principalement de certaines informations manquantes dans les dossiers. Nonobstant ces écueils, notre étude a retrouvé une fréquence hospitalière de la tuberculose à microscopie positive de 30,4% de l’ensemble des patients hospitalisés. La moyenne d’âge de la population d’étude était de 37,5 ± 14,9 Ces résultats sont superposables aux données retrouvées dans la littérature [5-8]. Ce qui vient appuyer l’assertion selon laquelle la tuberculose est une affection qui touche avec prédilection les jeunes [9] en pleine activité avec des répercussions socioéconomiques préjudiciables. Nous avons retrouvé un délai de prise en charge long en moyenne 111,8 ± 108,6 jours. Ce constat n’est pas particulier au Sénégal. Des travaux faits dans certains pays ont abouti à la même observation [7, 10, 11]. Ce délai de prise en charge long conditionne l’évolution et le pronostic la tuberculose [7]. Ce qui pourrait incontestablement expliquer non seulement l’importance des lésions extensives (71,2%) et bilatérales (45,5%), mais également le retentissement sur l’état général, car 52,2% des patients de l’étude avaient une dénutrition. Les rechutes tuberculeuses étaient plus fréquentes (26,1%) chez les fumeurs bien qu’étant non statistiquement significative. Ce résultat est concordant avec les résultats des autres études retrouvées dans la littérature [12, 13]. Cela pourrait s’expliquer par l’immunodépression des lymphocytes pulmonaires, la diminution de l’activité phagocytaire, de la bactéricide des macrophages alvéolaires et de la libération des TNF-a, IL-1, - 6, - 8, et 12 induits par la fumée de tabac [13-15] qui pourraient favorisés la réactivation du bacille de Koch (BK) rester quiescent dans les macrophages alvéolaires et favoriser ainsi une rechute tuberculeuse. L’aspect de poumon détruit (4,5%) avait été retrouvé du côté gauche dans la majorité des cas dans notre étude.

La prédominance du côté gauche à été décrit par plusieurs auteurs [16,17]. Cela pourrait s’expliquer par le fait que la bronche souche gauche est considérablement plus longue et plus étroite que la bronche souche droite et la région péri-bronchique gauche est limitée par la proximité de l’aorte. A cet effet, cette bronche est plus sujette à un mauvais drainage et à une compression extrinsèque par des adénopathies adjacentes d’où la vulnérabilité du poumon gauche à la chronicité des infections [16]. En présence d’une caverne, le diagnostic bactériologique de tuberculose est aisé, l’étude de l’expectoration étant positive dans 98% des cas à l’examen direct [18]. Plus de la moitié (59,1%) des patients de notre étude avait des lésions cavitaires associées à la présence de bacilles acido-alcoolo-résistants (BAAR). La lobite supérieure excavée représentait 7,6% des lésions dans notre étude. En effet, le siège de prédilection de la tuberculose pulmonaire est le segment postérieur d’un des lobes supérieurs ou le segment apical du lobe inférieur. C’est dans ces segments supérieurs que la pression partielle en oxygène est la plus élevée. Car, ces zones ne sont que très peu perfusées chez un homme au repos en position debout, alors que la ventilation y reste importante. Un rapport ventilation-perfusion très élevé explique que l’air alvéolaire soit très riche en oxygène et pauvre en gaz carbonique [19]. L’oxygène est indispensable au développement du bacille tuberculeux, germe à croissance lente aérobie strict. La tuberculose pulmonaire sur lésions séquellaires de tuberculose n’ont pas été en reste (4,5%). En l’absence de résultats des crachats, qui confortent le diagnostic de tuberculose, ces lésions sont complexes, difficiles d’interprétation en particulier lorsqu’elles sont étendues, requiert souvent la tomodensitométrie (TDM) du thorax pour détecter les aspects équivoques et analyser les complications [19]. Elle a prouvé son efficacité dans l’évaluation de la tuberculose [19].

## Conclusion

Le cliché radiologique occupe une place prépondérante dans le diagnostic de la tuberculose pulmonaire, surtout dans les pays en développement. Les lésions radiologiques de la tuberculose pulmonaire sont multiples, et pour la plupart aspécifiques. Dans les pays à forte prévalence tuberculeuse, nous préconisons chez un sujet jeune à fortiori fumeur, d’orienter la démarche étiologique vers la tuberculose pulmonaire devant les images radiologiques infiltratives associées ou non à des lésions cavitaires afin de raccourcir les délais diagnostic.

### Etat des connaissances actuelles sur le sujet

- L’infection à VIH/SIDA modifie les caractéristiques radiographiques de la tuberculose pulmonaire.

### Contribution de notre étude à la connaissance

- Peu d’étude ont été réalisée sur les particularités radiologiques de la tuberculose à microscopie positive à Dakar;
- Caractéristiques des patients chez qui orienté la démarche diagnostic étiologique vers une tuberculose pulmonaire;
- Orienter la démarche étiologique vers la tuberculose pulmonaire devant les anomalies radiologiques chez un sujet jeune et à fortiori fumeur.

## Conflits d’intérêts

Les auteurs ne déclarent aucun conflit d'intérêts.
